# Supplementary material for: Application of CoLD-CoP to Detecting Competitively and Cooperatively Binding Ligands
Source: Biomolecules. 2024 Sep 9;14(9):1136. doi: 10.3390/biom14091136 (PMC11430148; doi:10.3390/biom14091136)
Supplement: Supplementary file 1 [file biomolecules-14-01136-s001.zip › biomolecules-3108517-supplementary.pdf]

Table S1: Assigned Peaks in DOSY Spectrum of Ligand Mixture (with neither tyrosinase nor α-cyano-4-hydroxycinnamic acid present)

Chemical Shift	Intensity	Diffusion Coefficient	± Std. Error	Assignment	MW	Predicted MW	log(MW)	log(Diffusion Coeff)
1.97	0.04	5.41	0.01	GlcNAc	221.21	244.65	5.40	1.69
2.46	0.03	5.17	0.01	Thiamine	265.36	266.89	5.58	1.64
2.50	0.03	5.01	0.01	Thiamine	265.36	283.39	5.58	1.61
3.11	0.03	6.59	0.01	Tryptamine	160.22	167.66	5.08	1.89
3.26	0.02	6.76	0.01	Tryptamine	160.22	159.93	5.08	1.91
3.39	0.01	5.45	0.03	Thiamine	265.36	241.51	5.58	1.70
3.48	0.00	6.29	0.07	GlcNAc	221.21	183.55	5.40	1.84
3.66	0.39	6.98	0.01	Tris	121.14	150.34	4.80	1.94
3.76	0.01	5.44	0.03	GlcNAc	221.21	242.08	5.40	1.69
3.80	0.01	5.16	0.01	Thiamine	265.36	267.80	5.58	1.64
3.85	0.00	5.86	0.07	GlcNAc	221.21	210.18	5.40	1.77
4.30	0.02	6.60	0.02	Tartrate	148.07	167.53	5.00	1.89
4.70	1.13	20.93	0.02	Water	18.02	18.37	2.89	3.04
5.13	0.00	5.41	0.04	GlcNAc	221.21	244.88	5.40	1.69
5.41	0.02	4.97	0.02	Thiamine	265.36	288.16	5.58	1.60
6.88	0.01	7.90	0.03	Salicylate	138.12	118.61	4.93	2.07
6.92	0.00	7.82	0.06	Salicylate	138.12	120.87	4.93	2.06
7.21	0.01	6.77	0.03	Tryptamine	160.22	159.50	5.08	1.91
7.25	0.01	6.73	0.03	Tryptamine	160.22	161.24	5.08	1.91
7.39	0.01	7.80	0.04	Salicylate	138.12	121.57	4.93	2.05
7.46	0.01	6.74	0.03	Tryptamine	160.22	160.92	5.08	1.91
7.61	0.01	6.67	0.03	Tryptamine	160.22	163.86	5.08	1.90
7.74	0.01	7.71	0.04	Salicylate	138.12	124.21	4.93	2.04
7.93	0.01	5.28	0.02	Thiamine	265.36	256.25	5.58	1.66

Table S2: Assigned Peaks in DOSY Spectrum of Ligand+Protein Mixture (with tyrosinase but not α-cyano-4-hydroxycinnamic acid present)

Chemical Shift	Intensity	Diffusion Coeff	± Std. Error	Assignment	MW	Pred. MW	log(MW)	log(Diffusion Coeff)
1.97	0.01	5.57	0.01	GlcNAc	221.21	229.06	5.40	1.72
2.41	0.01	5.26	0.01	Thiamine	265.36	256.06	5.58	1.66
2.47	0.01	5.04	0.02	Thiamine	265.36	277.58	5.58	1.62
3.11	0.01	6.63	0.01	Tryptamine	160.22	163.40	5.08	1.89
3.26	0.01	6.90	0.02	Tryptamine	160.22	151.21	5.08	1.93
3.39	0.00	5.76	0.03	Thiamine	265.36	214.71	5.58	1.75
3.48	0.00	6.87	0.13	GlcNAc?		152.50		1.93
3.63	0.09	7.01	0.02	Tris	121.14	146.57	4.80	1.95
3.73	0.00	5.99	0.06	GlcNAc	221.21	199.18	5.40	1.79
3.79	0.00	5.51	0.02	Thiamine	265.36	233.59	5.58	1.71
3.85	0.00	7.50	0.28	GlcNAc?		128.43		2.02
4.24	0.00	7.20	0.04	Tartrate	148.07	139.28	5.00	1.97
4.70	1.12	21.19	0.01	Water	18.02	17.14	2.89	3.05
5.13	0.00	4.65	0.05	GlcNAc	221.21	324.43	5.40	1.54
5.34	0.00	5.05	0.02	Thiamine	265.36	276.73	5.58	1.62
6.88	0.00	7.25	0.12	Salicylate	138.12	137.43	4.93	1.98
7.21	0.00	6.89	0.03	Tryptamine	160.22	151.42	5.08	1.93
7.25	0.00	6.86	0.02	Tryptamine	160.22	152.70	5.08	1.93
7.38	0.00	7.32	0.17	Salicylate	138.12	134.67	4.93	1.99
7.46	0.00	6.88	0.03	Tryptamine	160.22	152.08	5.08	1.93
7.61	0.00	6.71	0.03	Tryptamine	160.22	159.66	5.08	1.90
7.75	0.00	6.77	0.16	Salicylate	138.12	156.99	4.93	1.91
7.96	0.00	5.17	0.03	Thiamine	265.36	264.40	5.58	1.64

Table S3: Assigned Peaks in DOSY Spectrum of Ligand+Protein Mixture (with both tyrosinase and α-cyano-4-hydroxycinnamic acid present)

Chemical Shift	Intensity	Diffusion Coeff	± Std. Error	Assignment	MW	Pred. MW	log(MW)	log(Diffusion Coeff)
1.97	0.01	5.41	0.01	GlcNAc	221.21	231.00	5.40	1.69
2.42	0.00	4.84	0.03	Thiamine	265.36	283.65	5.58	1.58
3.10	0.00	6.38	0.02	Tryptamine	160.22	170.46	5.08	1.85
3.26	0.00	6.77	0.02	Tryptamine	160.22	152.59	5.08	1.91
3.39	0.00	5.78	0.03	Thiamine	265.36	204.89	5.58	1.75
3.48	0.00	7.33	0.08	GlcNAc?		131.81		1.99
3.63	0.00	7.45	0.10	Tris	121.14	127.97	4.80	2.01
3.79	0.00	5.61	0.02	GlcNAc	221.21	216.35	5.40	1.72
3.85	0.00	7.73	0.20	GlcNAc?		119.49		2.05
4.25	0.00	7.24	0.02	Tartrate	148.07	134.99	5.00	1.98
4.70	1.13	21.18	0.01	Water	18.02	18.53	2.89	3.05
5.13	0.00	4.41	0.06	GlcNAc	221.21	337.18	5.40	1.48
6.88	0.00	7.69	0.09	Salicylate	138.12	120.63	4.93	2.04
6.92	0.00	6.24	0.03	HCCA	189.17	177.66	5.24	1.83
7.21	0.00	6.58	0.03	Tryptamine	160.22	161.06	5.08	1.88
7.24	0.00	6.64	0.02	Tryptamine	160.22	158.21	5.08	1.89
7.38	0.00	7.46	0.17	Salicylate	138.12	127.56	4.93	2.01
7.46	0.00	6.57	0.03	Tryptamine	160.22	161.50	5.08	1.88
7.61	0.00	6.58	0.03	Tryptamine	160.22	160.88	5.08	1.88
7.75	0.00	7.12	0.13	Salicylate	138.12	139.03	4.93	1.96
7.80	0.00	5.95	0.03	HCCA	189.17	193.66	5.24	1.78
7.94	0.00	6.07	0.03	HCCA	189.17	187.14	5.24	1.80

Abbreviations used in tables: Coeff: Coefficient; GlcNAc: N-Acetyl-Glucosamine; HCCA: α-cyano-4-hydroxycinnamic acid; MW: Molecular Weight or better Molecular Mass; Std. Error: Standard Error; Tris: tris(hydroxymethyl)aminomethane also known as tromethamine. The logarithm used is the natural logarithm. Diffusion coefficients are in units of 10^{-10} m^2 s^{-1}. Standard errors in diffusion coefficients are those estimated in least squares fitting of DOSY decay curves for each peak analyzed and typically underestimate the true uncertainty in diffusion coefficients inferred from DOSY data (c.f. references [14] and [15]).
